# Metabolic connectivity and memory in mesial temporal lobe epilepsy

**DOI:** 10.1093/braincomms/fcag365

**Published:** 2026-10-14

**Authors:** Ondřej Strýček, Pavel Říha, Martin Kojan, Eva Brichtová, Zuzana Hummelová, Zdeněk Řehák, Milan Brázdil

**Affiliations:** Brno Epilepsy Center, First Department of Neurology, St. Anne’s University Hospital and Faculty of Medicine, Masaryk University, Member of ERN-EpiCARE, Brno 60300, Czech Republic; Central European Institute of Technology (CEITEC), Masaryk University, Brno 62500, Czech Republic; Central European Institute of Technology (CEITEC), Masaryk University, Brno 62500, Czech Republic; Brno Epilepsy Center, First Department of Neurology, St. Anne’s University Hospital and Faculty of Medicine, Masaryk University, Member of ERN-EpiCARE, Brno 60300, Czech Republic; Central European Institute of Technology (CEITEC), Masaryk University, Brno 62500, Czech Republic; Brno Epilepsy Center, Department of Neurosurgery, St. Anne’s University Hospital, Faculty of Medicine, Masaryk University, Member of the ERN EpiCARE, Brno 60300, Czech Republic; Brno Epilepsy Center, Department of Neurosurgery, St. Anne’s University Hospital, Faculty of Medicine, Masaryk University, Member of the ERN EpiCARE, Brno 60300, Czech Republic; Department of Nuclear Medicine, Masaryk Memorial Cancer Institute, Brno 60200, Czech Republic; Brno Epilepsy Center, First Department of Neurology, St. Anne’s University Hospital and Faculty of Medicine, Masaryk University, Member of ERN-EpiCARE, Brno 60300, Czech Republic; Central European Institute of Technology (CEITEC), Masaryk University, Brno 62500, Czech Republic

**Keywords:** mesial temporal lobe epilepsy, positron emission tomography, metabolic connectivity, memory

## Abstract

Memory decline after anterior mesial temporal resection in mesial temporal lobe epilepsy with hippocampal sclerosis (MTLE-HS) remains incompletely explained by structural and clinical factors, suggesting that networks may contribute to cognitive vulnerability. We investigated whether presurgical metabolic connectivity (MC), derived from fluorodeoxyglucose positron emission tomography (FDG-PET) and reflecting metabolic network organization, is associated with memory performance and postsurgical cognitive outcome in MTLE-HS. We studied 83 patients with MTLE-HS who underwent anterior mesial temporal resection. All had interictal FDG-PET and pre- and postsurgical neuropsychological testing. MC was computed using a normative connectivity framework and examined in relation to memory performance, analysed separately for left and right TLE. The effect of resection extent on cognitive outcome was also assessed. Presurgical analyses revealed distinct lateralized patterns. In right temporal lobe epilepsy (RTLE), immediate visual memory correlated positively with MC across widespread fronto–cingulate–temporal networks, frequently including the lesional hippocampus. In left temporal lobe epilepsy (LTLE), immediate verbal memory showed only negative correlations with extratemporal MC. In RTLE, presurgical MC also correlated with postsurgical change in immediate visual memory. Predominantly negative associations involved ipsilateral hippocampal–cingulate and fronto-temporal pathways, whereas positive correlations were confined to contralateral posterior cingulate connections with frontal and temporal regions. In LTLE, no significant correlations with postsurgical memory change were identified. Extent of resection did not correlate with cognitive outcome. MC was significantly associated with memory performance in MTLE-HS, showing clear lateralized differences. In RTLE, presurgical MC was associated with postsurgical outcome: ipsilateral hippocampal–cingulate connectivity was linked to decline, whereas contralateral posterior cingulate pathways supported preservation. No such associations were found in LTLE, consistent with more constrained network reorganization within left-lateralized verbal memory systems. These effects were independent of resection extent, indicating that memory prognosis after AMTR in RTLE may depend on network-level mechanisms and that FDG-PET-derived MC could serve as a biomarker for cognitive risk assessment.

## Teaser

Strýček *et al*. report that positron emission tomography-derived metabolic connectivity is associated with memory performance in mesial temporal lobe epilepsy with hippocampal sclerosis. Presurgical metabolic network patterns predicted postoperative memory change, particularly after right-sided surgery, supporting network-based cognitive risk assessment.

## Graphical Abstract

**Graphical Abstract:**
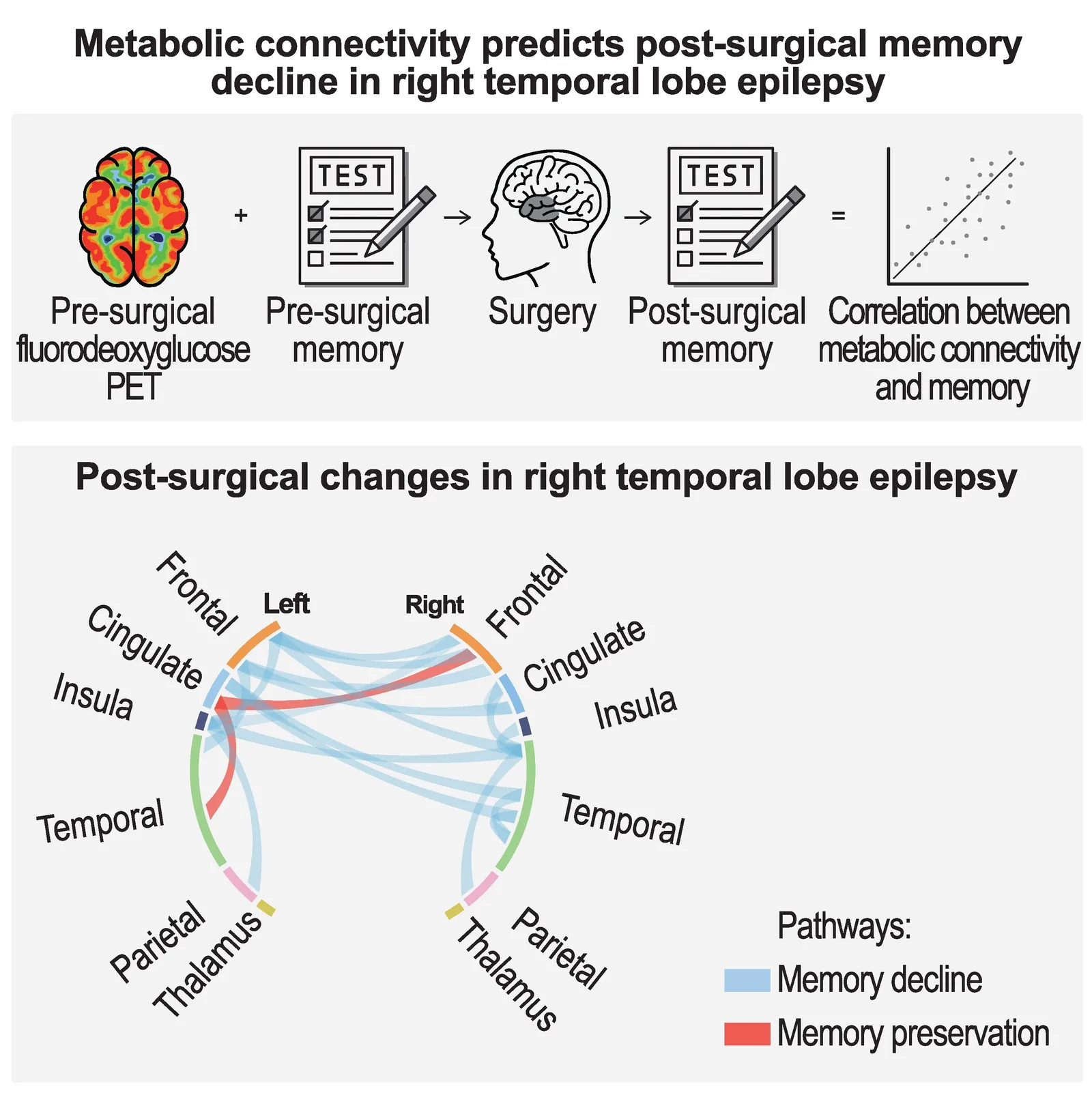

## Introduction

Mesial temporal lobe epilepsy (MTLE) with hippocampal sclerosis (HS) is the most prevalent drug-resistant epilepsy (DRE) in adults.^1,2^ Surgical intervention, specifically anterior mesial temporal resection (AMTR), represents a crucial treatment option for these patients. However, complete seizure freedom is achieved in ∼57% of patients.^2^ This suggests that epileptogenic networks often extend beyond the resected hippocampal lesion.^3-5^ A substantial proportion of patients experience significant memory decline following AMTR; therefore the identification of predictive biomarkers is critical to minimize cognitive deficits while ensuring effective seizure control.^6^

Network-based approaches have shown that memory impairment in MTLE may arise from alterations in broader brain networks, not only from hippocampal pathology.^7^ Functional connectivity (FC) studies based on resting-state and task-based fMRI (fMRI-FC) have demonstrated that altered connectivity in regions distant from the resection site or the hippocampus could influence postsurgical memory decline.^7-10^

Fluorodeoxyglucose positron emission tomography (FDG-PET) is a nuclear imaging method that provides an indirect *in vivo* measure of regional neuronal and synaptic activity through assessment of cerebral glucose metabolism.^11^ Unlike fMRI, which indirectly measures neuronal activity through blood oxygenation level-dependent signal fluctuations, FDG-PET provides a metabolic perspective on brain function.^12^ It is a well-established tool in epilepsy surgery planning, with the extent of hypometabolism—within the lesion and surrounding area, or even extratemporally—serving as a predictor of surgical outcome.^13,14^ Moreover, temporal hypometabolism has been associated with the degree of postsurgical memory decline.^15,16^ In contrast, no consistent relationship has been demonstrated between extratemporal hypometabolism and memory change following surgery.^17,18^

However, as memory is network-based, we hypothesized that altered PET metabolism in extratemporal regions may also contribute to postsurgical memory outcomes. In this context, metabolic connectivity (MC), a relatively novel method derived from FDG-PET data, has already been shown to predict surgical outcome in epilepsy at the network level and may also provide markers of cognitive decline.^4,19^ MC may represent a meaningful approach to characterize alterations in the memory network.

This study investigates the correlation of MC with pre- and postsurgical memory performance. We aimed to determine whether FDG-PET-derived MC predicts presurgical memory performance and postsurgical memory change in MTLE-HS, independently of resection extent. We hypothesized that presurgical MC captures lateralized organization of memory networks and that, beyond association with baseline performance, these network patterns may predict postoperative cognitive change.

## Materials and methods

### Participants

We included 83 patients with drug-resistant MTLE and histopathologically confirmed HS who underwent AMTR at the Brno Epilepsy Center between 2009 and 2023. The cohort consisted of 31 men and 52 women. Patients were selected consecutively from the local surgical database without prior selection or exclusion, based solely on the availability of complete presurgical and postsurgical data that met the neuropsychological and imaging criteria described below. No patient had a documented history or clinical suspicion of autoimmune encephalitis. Of the total cohort, 44 patients had left temporal lobe epilepsy (LTLE) and 39 had right temporal lobe epilepsy (RTLE).

All patients underwent interictal FDG-PET and high-resolution 3D T_1_-weighted MRI scans acquired on a Siemens 3T scanner. Presurgical neuropsychological assessments were performed within a maximum of 6 months of PET acquisition. Postsurgical neuropsychological assessments were available for 29 LTLE and 17 RTLE patients, performed between 12 and 18 months after surgery; patients with incomplete or out-of-window data were excluded from analyses. The neuropsychological protocol focused on visual and verbal memory performance, covering immediate and delayed recall of both verbal and visual material.

Results were expressed as standardized Memory Quotients (MQs) derived from Wechsler-based scales, using the Czech version of the Wechsler Memory Scale–III.^20^ This retrospective study was approved by the Institutional Ethics Committee and conducted in accordance with the Declaration of Helsinki. The requirement for individual informed consent was waived due to the retrospective design and use of anonymized data.

The control group (CG) consisted of 25 individuals (14 women, 11 men; mean age 53 ± 18 years) drawn from an oncology database. All underwent standard FDG-PET as part of routine oncology screening and had normal findings. They had no evidence of intracranial or head–neck tumours, no neurological or psychiatric disorders, had no pathological findings on MRI, and were not taking medications affecting brain metabolism.

### Neuroimaging data

#### FDG-PET acquisition and preprocessing

Patients were imaged as outpatients in the interictal state. No patient reported seizures on the day of the PET scan or during the PET acquisition. All subjects were instructed to fast for 6 h before the scan and to rest in a quiet, darkened room for 45–60 min after FDG administration. The administered dose of FDG was between 170 and 200 MBq per subject, with no weight differentiation. The PET images were acquired using a Siemens mCT Flow PET/CT scanner in 3D mode following the ‘Brain’ protocol. Low-dose CT was used for attenuation correction.

Preprocessing was performed in SPM12 (Wellcome Department of Cognitive Neurology, University College London, UK). All FDG-PET images were spatially normalized into standard Montreal Neurological Institute (MNI) stereotactic space using an in-house FDG-PET template generated according to previously published procedures.^4^ Normalized images were smoothed with an isotropic Gaussian kernel of 8 mm FWHM. Regional standardized uptake value ratios were calculated using the cerebellum as the reference region.

#### MRI assessment of HS

MRI characteristics were assessed using a protocol adapted from Chassoux *et al*.^21^ Two experienced neurologists, blinded to clinical outcomes, visually evaluated the presurgical MRI scans to determine the degree of HS (classified as mild, moderate, or severe), the presence of temporal pole abnormalities (signal changes or gray–white matter blurring), and the extent of temporal lobe atrophy.

#### Resection extent

To assess the potential influence of resection extent on cognitive outcomes, presurgical and postsurgical high-resolution MRI datasets were spatially normalized to the MNI space. Specifically, postsurgical images were first rigidly coregistered to the corresponding presurgical scans. The presurgical images were then normalized to MNI space using SPM12, and the resulting non-linear deformation fields were subsequently applied to the coregistered postsurgical images. Following the standardized protocol described by Sone *et al*.,^6^ the anterior end of the temporal lobe and the posterior hippocampal tail were identified, the hippocampal axis was defined and the anterior–posterior lengths of both the hippocampus and temporal lobe were measured. Resection lengths were determined by comparing pre- and postsurgical images along the same axis and were expressed as a percentage of the original structure’s length. Only linear anterior–posterior measurements were used; resection volumes were not assessed.

### Statistical analysis

#### Participant characteristics

Demographic characteristics (including number of patients, sex, age, epilepsy duration, seizure frequency, epilepsy history, and outcome) were collected for all patients and summarized separately for the total cohort as well as for the LTLE and RTLE subgroups. Group comparisons between LTLE and RTLE were performed for categorical variables using chi-square tests, with significance set at *P* < 0.05. Inter-rater agreement for visual assessment of MRI abnormalities was assessed using quadratic weighted Cohen’s kappa, appropriate for ordinal rating scales.

#### Memory evaluation

Presurgical and postsurgical MQ scores were analysed for verbal and visual memory performance in both immediate and delayed recall conditions. Postsurgical change (ΔMQ) was defined as the difference between postsurgical and presurgical scores. Clinically significant decline was defined as *a* ≥ 1 SD (15-point) decrease from the presurgical score.

#### Metabolic connectivity evaluation

Regional FDG-PET values were extracted from memory-related regions of interest (ROIs) defined using a modified Automated Anatomical Labeling atlas (AAL1).^7,8,22-26^ Individual MC matrices were constructed using the approach proposed by Huang *et al*.^19^ and previously applied in our work.^4^ In brief, a group-level reference connectivity matrix was first estimated from the CG based on interregional correlations of FDG-PET values. For each patient, regional deviations in FDG-PET from the CG mean were quantified and used to derive a subject-specific weighting matrix reflecting interregional differences in metabolic abnormality. This weighting matrix was then applied element-wise to the CG reference connectivity matrix, yielding an individualized MC matrix that preserves the normative network topology while modulating connection strengths according to patient-specific metabolic deviations. A detailed mathematical formulation and computational workflow have been described elsewhere.^4,27^

The selection of ROIs was guided by their established involvement in memory networks, particularly in the context of MTLE. Key structures included the hippocampus, fusiform gyrus, temporal pole, lateral temporal cortex, dorsolateral prefrontal cortex, orbitofrontal cortex, insula, lateral parietal cortex, cingulate cortex, and thalamus. These regions represent core hubs of the distributed episodic memory network and have been implicated both in pathological processes related to seizure generation and in compensatory reorganization mechanisms.^7-9,22-26,28-30^ A detailed list of ROIs, along with justification for their inclusion, is provided in the Supplementary material.

#### Correlation of metabolic connectivity and memory scores

We investigated the correlation between MC and both presurgical and postsurgical MQ scores, separately for the left and right TLE groups. The cognitive outcome was defined as the ΔMQ. Given the lateralized memory specialization, we analysed RTLE patients in relation to visual MQ and LTLE patients in relation to verbal MQ, which are considered the most clinically relevant for each group. As evidence indicates high concordance and limited discriminative value between immediate and delayed recall measures in TLE, we restricted our primary analyses to immediate MQ.^31^

All statistical analyses accounted for relevant clinical covariates including sex, age, epilepsy duration, age at onset, imaging scanner type, and MRI characteristics (degree of HS, temporal polar abnormality, and temporal atrophy). To control these covariates, both MC measures and memory quotient scores were first adjusted using generalized linear models, and residualized values were subsequently used for correlation analyses. Associations between covariate-adjusted variables were assessed using Pearson correlation coefficients. *P*-values were corrected for multiple comparisons using the false discovery rate (FDR), with FDR-corrected *P*-values < 0.05 considered statistically significant.

As an exploratory secondary analysis, we also tested whether presurgical MC was associated with postoperative seizure outcome, defined as seizure freedom (ILAE class 1) versus non-seizure-free outcome (ILAE classes 2–5), separately in LTLE and RTLE.

#### Correlation of resection extent with memory outcomes

In addition, we examined whether the extent of surgical resection was associated with postsurgical cognitive change (ΔMQ) in order to account for potential structural effects on memory outcome. Memory scores were adjusted for the same covariates and using the same procedure as in the analyses of MC and memory scores, whereas resection extent was not adjusted. These associations were assessed using Pearson correlation coefficients, with statistical significance set at *P* < 0.05.

## Results

### Clinical and neuroimaging data characteristics

Demographic, clinical, seizure-related, and MRI characteristics of the cohort are summarized in Table 1. No significant differences were observed between LTLE and RTLE groups regarding age, sex distribution, epilepsy duration, age at onset, seizure frequency, number of ASMs, FBTCS occurrence, or seizure outcome. MRI features were comparable between groups for HS degree and temporal polar abnormality; however, temporal lobe atrophy was more frequent in LTLE than in RTLE (45% versus 13%, *P* = 0.0027). Inter-rater agreement for visual assessment of MRI abnormalities was high, with a quadratic weighted Cohen’s kappa of 0.84 and exact agreement in 80.7% of cases.

**Table 1:** Characteristics of patients

Clinical characteristics	TLE	LTLE	RTLE	Left versus right (*P-*value)
**Number**	83	44	39	
**Sex,** n males/females	31/52	19/25	12/27	0.3475
**Age at epilepsy onset,** years mean ± SD	14 ± 11	14 ± 12	14 ± 10	0.8920
**Age at surgery,** years mean ± SD	40 ± 11	38 ± 11	42 ± 11	0.1678
**Epilepsy duration,** years mean ± SD	26 ± 15	25 ± 15	28 ± 15	0.2804
**Seizure frequency,** n/month	9 ± 10	8 ± 8	9 ± 11	0.5275
**FBTCS,** n of patients (%)	47 (57%)	25 (56%)	22 (56%)	1.0000
**Epilepsy history,** n of patients (%)				
Familial epilepsy	7 (8%)	4 (9%)	3 (8%)	0.3828
Early brain injury	16 (20%)	6 (14%)	10 (26%)	
Febrile seizures	20 (24%)	12 (27%)	8 (21%)	
**ASM** (mean ± SD)	2.3 ± 0.7	2.3 ± 0.7	2.3 ± 0.8	0.8382
**Outcome,** n of patients (%)				
ILAE 1	51 (61%)	28 (64%)	23 (59%)	0.8340
ILAE non-1	32 (39%)	16 (36%)	16 (41%)	
**MRI characteristics**				
HS degree (mean ± SD)	1.7 ± 0.9	1.8 ± 0.9	1.6 ± 0.9	0.2180
Temporal polar abnormality (n, %)	20 (24%)	10 (23%)	10 (26%)	0.9580
Temporal atrophy (n, %)	25 (30%)	20 (45%)	5 (13%)	**0**.**0027**

Table summarizes the demographic, clinical, seizure-related, and MRI characteristics of the study cohort, showing largely comparable demographic and clinical characteristics between patients with left and right temporal lobe epilepsy, with a significant difference in temporal lobe atrophy. Structural data characteristics of the patients are based on Chassoux *et al*.^19^ Significant differences are depicted in bold.

TLE, temporal lobe epilepsy; RTLE, right temporal lobe epilepsy; LTLE, left temporal lobe epilepsy; HS, hippocampal sclerosis; *n*, number; SD, standard deviation; y, years; FBTCS, focal to bilateral tonic-clonic seizure; ASM, anti-seizure medication.

Postsurgical cognitive outcome, expressed as ΔMQ, showed a clinically significant decline (≥15 points in MQ) in 34% of LTLE patients for immediate verbal memory, compared with 12% in RTLE patients. For immediate visual memory, significant decline occurred in 21% of LTLE and 12% of RTLE patients. Detailed presurgical and postsurgical neuropsychological results for verbal and visual modalities (immediate and delayed recall) are summarized in Table 2 and Fig. 1.

**Figure 1:**
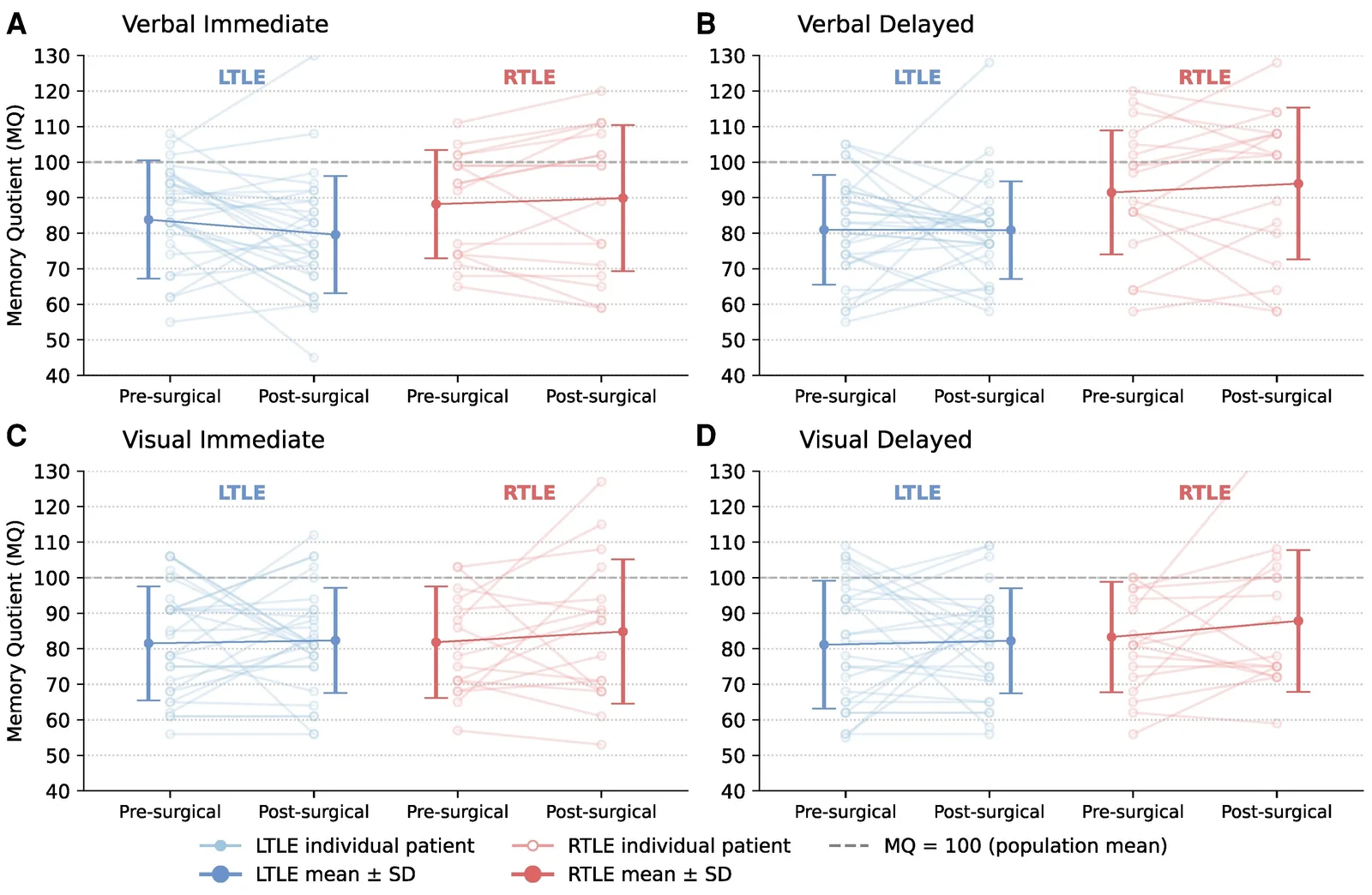
**Individual pre- and postsurgical memory changes in LTLE and RTLE.** Individual pre- and postsurgical memory changes in left and right temporal lobe epilepsy patients. Individual memory quotient (MQ) scores are shown before and after surgery for verbal immediate memory (**A**), verbal delayed memory (**B**), visual immediate memory (**C**), and visual delayed memory (**D**). Thin lines connect pre- and postsurgical scores for individual patients, and thicker lines with error bars indicate group mean ± standard deviation (SD). Blue denotes patients with left temporal lobe epilepsy (LTLE), and red denotes patients with right temporal lobe epilepsy (RTLE). The dashed horizontal line indicates the normative population mean (MQ = 100). No inferential statistical testing was performed for this descriptive figure. The experimental unit was the individual patient. MQ, memory quotient; SD, standard deviation; LTLE, left temporal lobe epilepsy; RTLE, right temporal lobe epilepsy.

**Table 2:** Neuropsychological assessment of patients

MQ	LTLE presurgical (mean ± SD)	LTLE postsurgical (mean ± SD)	LTLE Δ (mean)	LTLE decline (≥ 15) (%)	RTLE presurgical (mean ± SD)	RTLE postsurgical (mean ± SD)	RTLE Δ (mean)	RTLE decline (≥ 15) (%)
**Verbal immediate**	84 ± 17	80 ± 17	−4	34	88 ± 15	90 ± 21	2	12
**Verbal delayed**	81 ± 15	81 ± 14	0	21	91 ± 17	94 ± 21	2	18
**Visual immediate**	82 ± 16	82 ± 15	1	21	82 ± 16	85 ± 20	3	12
**Visual delayed**	81 ± 18	82 ± 15	1	21	83 ± 16	88 ± 20	5	6

Table summarizes presurgical and postsurgical verbal and visual memory performance in patients with left and right temporal lobe epilepsy, including mean changes and the proportion of patients with clinically significant memory decline. Δ = postsurgical − presurgical MQ; negative values indicate decline. A clinically significant change was defined as a decrease of ≥ 1 SD from baseline MQ, corresponding to ∼≥ 15 points on the MQ scale. Both Δ values and percentages of patients with ≥15-point decline were derived from individual paired differences, not from group means.

RTLE, right temporal lobe epilepsy; LTLE, left temporal lobe epilepsy; SD, standard deviation; MQ, memory quotient.

In an exploratory secondary analysis, presurgical MC did not differ according to postoperative seizure outcome. No significant MC differences between seizure-free patients (ILAE class 1) and non-seizure-free patients (ILAE classes 2–5) were identified in either LTLE or RTLE after correction for multiple comparisons.

### Correlations with presurgical memory

We first investigated whether presurgical MC patterns were associated with baseline memory performance and whether these associations differed between LTLE and RTLE patients. We identified both positive and negative correlations between MC and presurgical immediate MQ. In the presurgical analyses, a positive correlation indicates that higher MC is associated with better memory performance (higher MQ), while a negative correlation means that higher MC is associated with lower MQ.

#### Right temporal lobe epilepsy

In RTLE patients (*n* = 39), we found that presurgical immediate visual MQ positively correlated with MC in 24 connections. These were most often cross-hemispheric (10/24), followed by ipsihemispheric (8/24) and contrahemispheric (6/24). Connections typically involved the DLPFC, mesiotemporal structures, the cingulate cortex, and the temporal lateral cortex. Further significant connections were observed with the temporal pole, the lateral parietal cortex, and the insula. Regarding the lesional hippocampus, it was present in 9 of 24 significant connections, including both ipsilateral and contralateral, particularly with the DLPFC and temporal lateral cortex bilaterally, as well as with the insula and angular gyrus.

#### Left temporal lobe epilepsy

In LTLE patients (*n* = 44), presurgical immediate verbal MQ negatively correlated with MC in six connections. Most were cross-hemispheric (5/6) and one was contrahemispheric; no ipsihemispheric associations were detected. The most frequent regions were the temporal lateral cortex and the cingulate cortex, with additional connections involving the lateral parietal cortex, the temporal pole, and the fusiform gyrus. No significant connections involved the lesional hippocampus. Significant correlations of MC with presurgical immediate MQ in RTLE and LTLE are illustrated by chord diagrams in Figs 2 and 3. The corresponding correlation matrices are shown in Figs 4 and 5.

**Figure 2:**
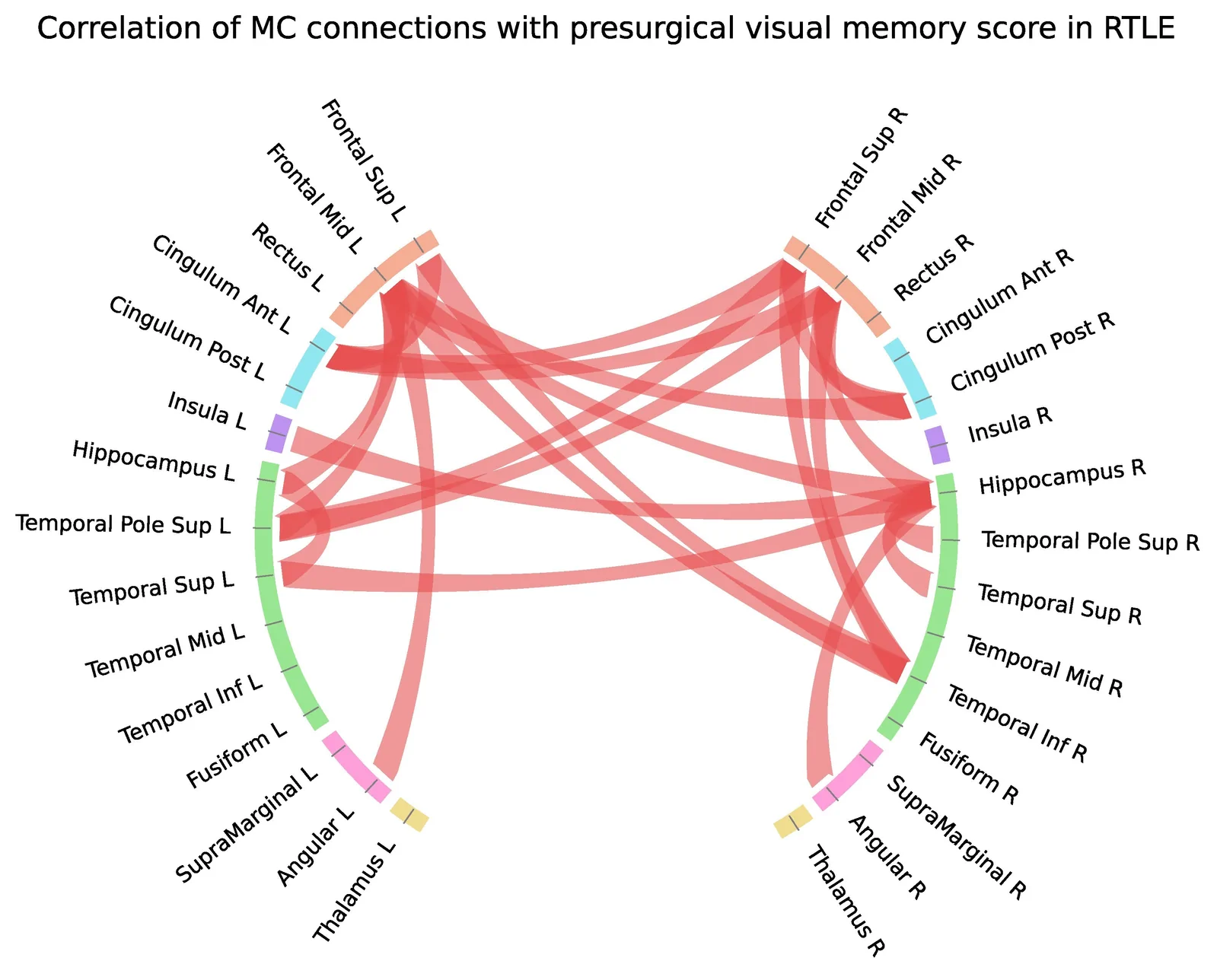
**Chord diagram of metabolic connectivity correlations with presurgical memory function in right temporal lobe epilepsy.** Chord diagram shows significant correlations between metabolic connectivity (MC) and presurgical immediate visual memory in right temporal lobe epilepsy. Red chords indicate positive correlations. Cortical and subcortical regions are colour-coded according to lobar affiliation (yellow = frontal, orange = cingulate, grey = insula, green = temporal, purple = parietal, violet = thalamus). Statistical analysis was performed using Pearson correlation across individual RTLE patients (*n* = 39), followed by false discovery rate correction across connections. Only significant connections are shown (FDR-corrected *P* < 0.05). The experimental unit was the individual patient. Exact *r* and *P-*values are provided in Supplementary Table 1. L, left; R, right; MC, metabolic connectivity; RTLE, right temporal lobe epilepsy. Abbreviations of anatomical regions are provided in the Supplementary material.

**Figure 3:**
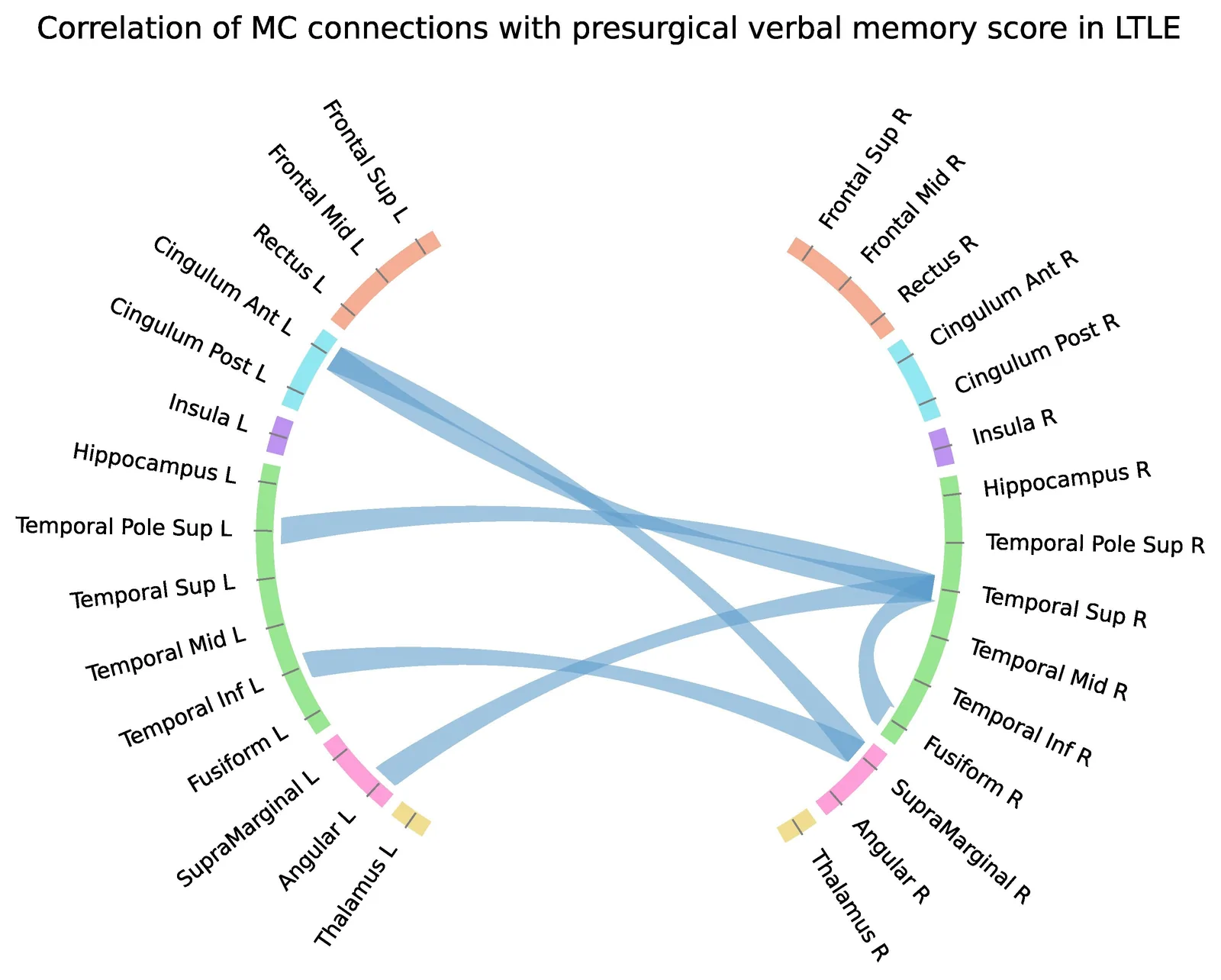
**Chord diagram of metabolic connectivity correlations with presurgical memory function in left temporal lobe epilepsy.** Chord diagram shows significant correlations between metabolic connectivity (MC) and presurgical immediate verbal memory in left temporal lobe epilepsy. Blue chords represent negative correlations. Cortical and subcortical regions are colour-coded according to lobar affiliation (yellow = frontal, orange = cingulate, grey = insula, green = temporal, purple = parietal, violet = thalamus). Statistical analysis was performed using Pearson correlation across individual LTLE patients (*n* = 44), followed by false discovery rate correction across connections. Only significant connections are shown (FDR-corrected *P* < 0.05). The experimental unit was the individual patient. Exact *r* and *P-*values are provided in Supplementary Table 1. L, left; R, right; MC, metabolic connectivity; LTLE, left temporal lobe epilepsy. Abbreviations of anatomical regions are provided in the Supplementary material.

**Figure 4:**
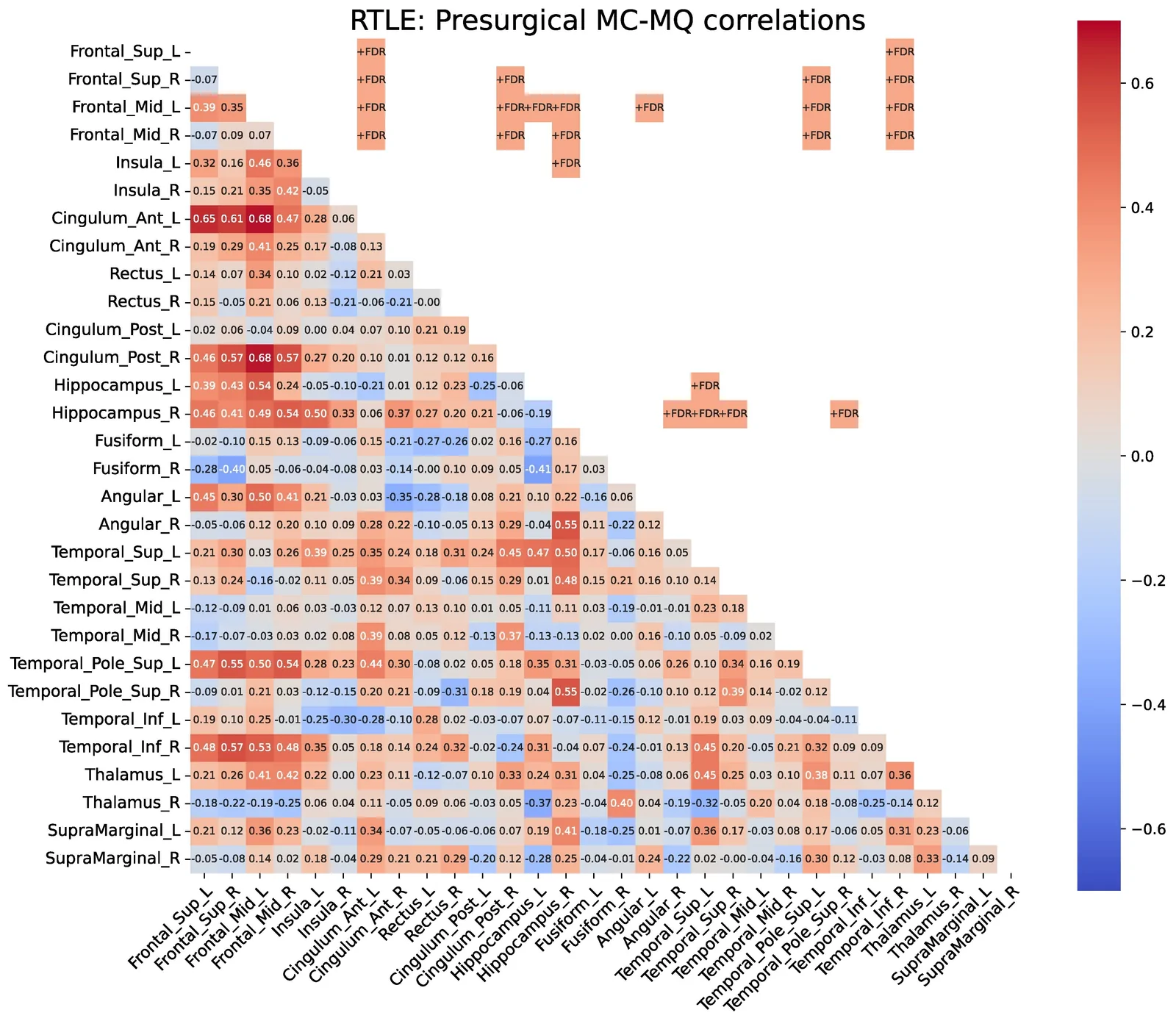
**Correlation of metabolic connectivity with presurgical memory function in right temporal lobe epilepsy.** Significant correlations between metabolic connectivity (MC) and immediate visual memory scores prior to surgery in right temporal lobe epilepsy. In each matrix, the lower left triangle depicts the strength and direction of correlation between MC (AAL regions of interest) and visual MQ. Warm colours indicate positive correlations (higher MC associated with better memory); cold colours indicate negative correlations (higher MC associated with poorer memory). Statistical analysis was performed using Pearson correlation across individual RTLE patients (*n* = 39), followed by false discovery rate correction across connections. The lower left triangle depicts Pearson *r* values, and the upper right triangle indicates statistically significant results (FDR-corrected *P* < 0.05). The experimental unit was the individual patient. Exact *r* and *P-*values are provided in Supplementary Table 1. L, left; R, right; MC, metabolic connectivity; MQ, memory quotient; FDR, false discovery rate; RTLE, right temporal lobe epilepsy. Abbreviations of anatomical regions are provided in the Supplementary material.

**Figure 5:**
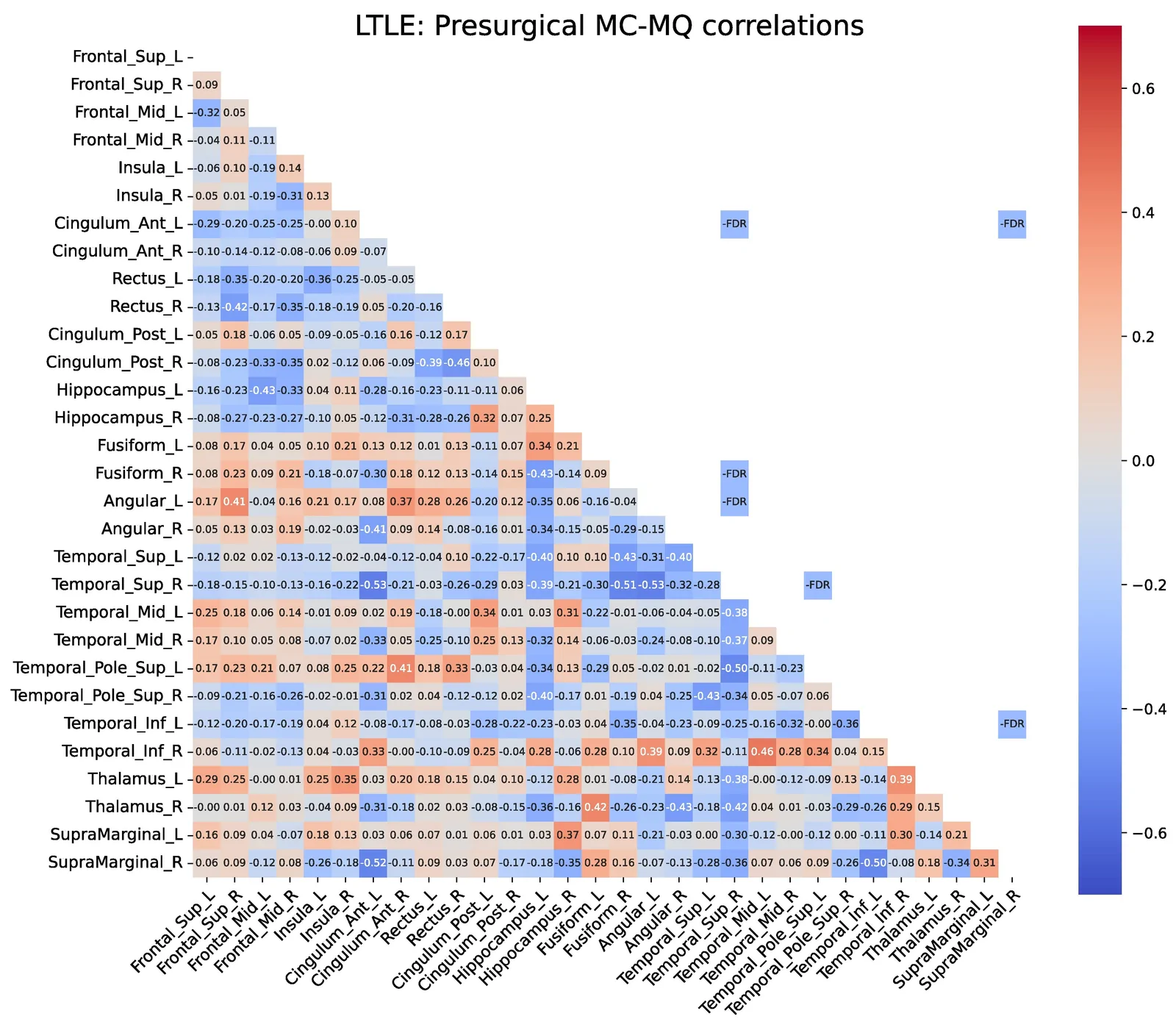
**Correlation of metabolic connectivity with presurgical memory function in left temporal lobe epilepsy.** Significant correlations between metabolic connectivity and immediate verbal memory scores prior to surgery in left temporal lobe epilepsy. In each matrix, the lower left triangle depicts the strength and direction of correlation between MC (AAL regions of interest) and verbal MQ. Warm colours indicate positive correlations; cold colours indicate negative correlations. Statistical analysis was performed using Pearson correlation across individual LTLE patients (*n* = 44), followed by false discovery rate correction across connections. The lower left triangle depicts Pearson *r* values, and the upper right triangle indicates statistically significant results (FDR-corrected *P* < 0.05). The experimental unit was the individual patient. Exact *r* and *P-*values are provided in Supplementary Table 1. L, left; R, right; MC, metabolic connectivity; MQ, memory quotient; FDR, false discovery rate; LTLE, left temporal lobe epilepsy. Abbreviations of anatomical regions are provided in the Supplementary material.

### Correlations with postsurgical memory

We next assessed whether presurgical MC was associated with postsurgical memory outcome and whether these relationships differed by epilepsy laterality. We identified both positive and negative correlations. Positive correlations indicate that higher presurgical MC was associated with higher postsurgical ΔMQ, reflecting a better outcome with less memory decline. Negative correlations indicate that higher presurgical MC was associated with lower postsurgical ΔMQ, reflecting a worse outcome with greater memory decline.

#### Right temporal lobe epilepsy

In RTLE patients with available postsurgical testing (*n* = 17), we found 20 connections in which presurgical MC correlated with postsurgical change in immediate visual MQ. Negative correlations predominated (18/20), with only two positive associations. Connections were most often cross-hemispheric (10/20), followed by ipsihemispheric (6/20) and contrahemispheric (4/20). The most frequent regions were the cingulate cortex, the mesiotemporal structures, the DLPFC, the insula, and the temporal lateral cortex. Additional associations were observed with the orbitofrontal cortex. Regarding the lesional hippocampus, it was present in 6 of 20 significant connections, all negative, including both ipsilateral and contralateral with frontal and temporal regions. The cingulate cortex was involved in 7 of 20 connections, mostly negative. Two positive correlations with memory performance were identified: between MC of the left posterior cingulum and the right middle frontal gyrus, and between MC of the left posterior cingulum and the left middle temporal gyrus.

#### Left temporal lobe epilepsy

In LTLE patients (*n* = 29), no significant connections were found between presurgical MC and postsurgical change in immediate verbal MQ.

Significant correlations of MC with postsurgical immediate MQ in RTLE are shown in Fig. 6, with the corresponding correlation matrix presented in Fig. 7. In LTLE, no significant correlations were identified; for completeness, the corresponding correlation matrix is provided in Supplementary Fig. 1. Complete numerical results of all significant MC–MQ correlations, including correlation coefficients, *P*-values, and mean differences from CG, are provided in Supplementary Table 1.

**Figure 6:**
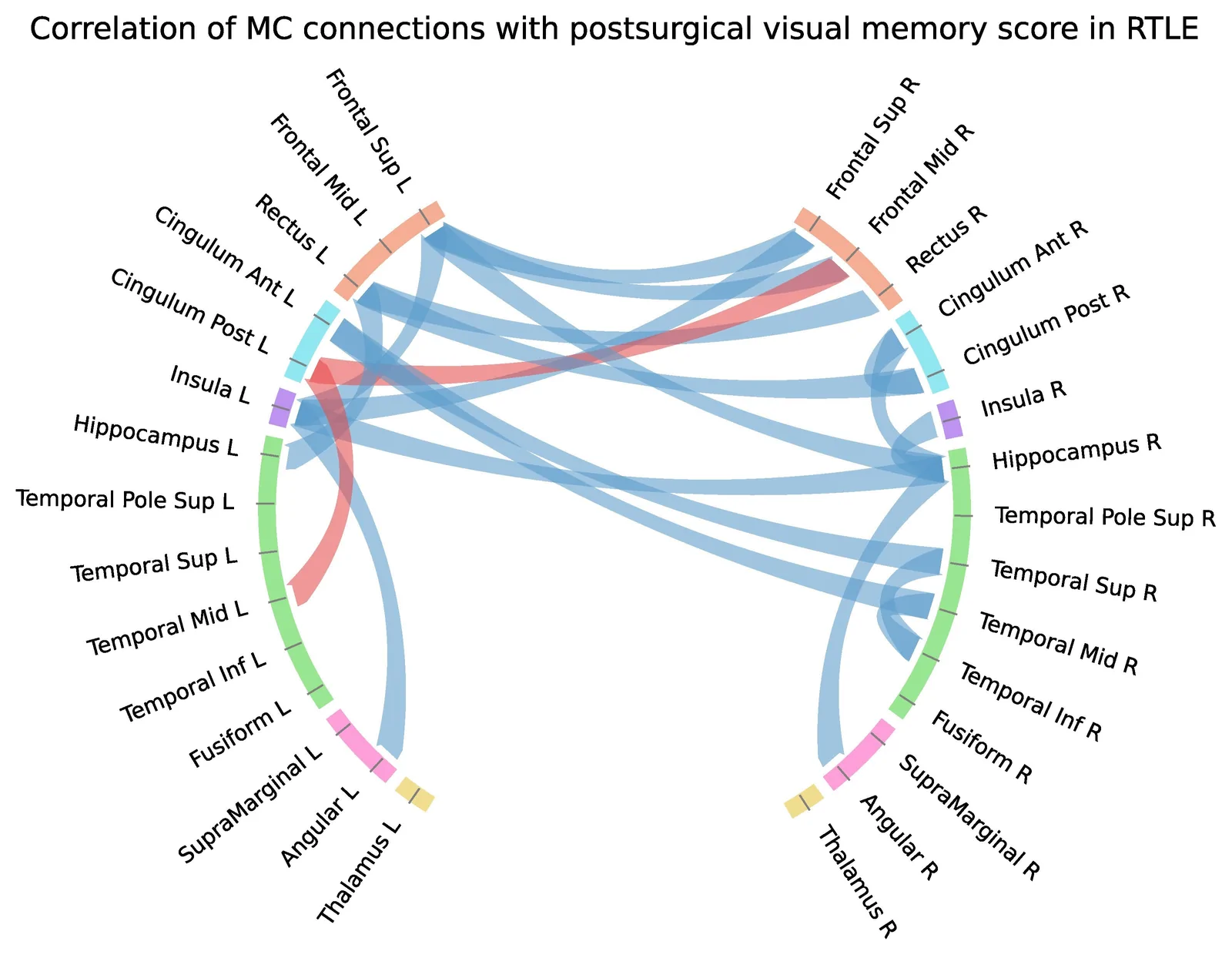
**Chord diagram of metabolic connectivity correlations with postsurgical memory change in right temporal lobe epilepsy.** Chord diagram shows significant correlations between metabolic connectivity (MC) and postsurgical change in immediate visual memory. Red chords represent positive correlations (greater MC associated with better postsurgical outcome), blue chords negative correlations (greater MC associated with worse outcome). Cortical and subcortical regions are colour-coded according to lobar affiliation (yellow = frontal, orange = cingulate, grey = insula, green = temporal, purple = parietal, violet = thalamus). Statistical analysis was performed using Pearson correlation across individual RTLE patients with available postsurgical testing (*n* = 17), followed by false discovery rate correction across connections. Only significant connections are shown (FDR-corrected *P* < 0.05). The experimental unit was the individual patient. Exact *r* and *P-*values are provided in Supplementary Table 1. L, left; R, right; MC, metabolic connectivity; RTLE, right temporal lobe epilepsy. Abbreviations of anatomical regions are provided in the Supplementary material.

**Figure 7:**
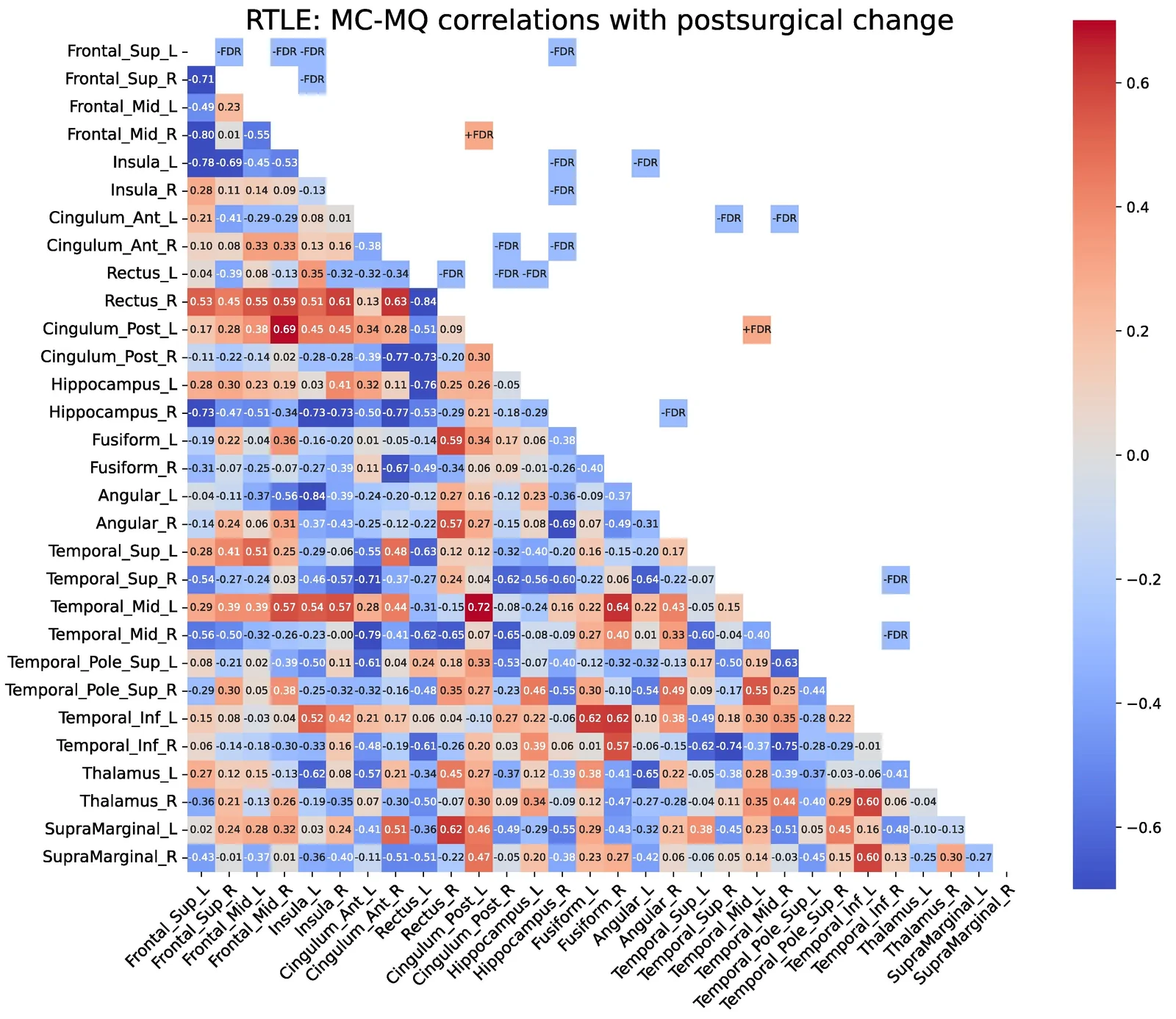
**Correlation of metabolic connectivity with postsurgical memory change in right temporal lobe epilepsy.** Significant correlations between metabolic connectivity (MC) and postsurgical change in visual immediate memory score in right temporal lobe epilepsy. Positive values indicate improved memory performance after surgery. The lower left triangle shows the correlation strength and direction (warm = positive, cold = negative). Statistical analysis was performed using Pearson correlation across individual RTLE patients with available postsurgical testing (*n* = 17), followed by false discovery rate correction across connections. The lower left triangle depicts Pearson *r* values, and the upper right triangle indicates statistically significant results (FDR-corrected *P* < 0.05). The experimental unit was the individual patient. Exact *r* and *P-*values are provided in Supplementary Table 1. L, left; R, right; MC, metabolic connectivity; MQ, memory quotient; FDR, false discovery rate; RTLE, right temporal lobe epilepsy. Abbreviations of anatomical regions are provided in the Supplementary material.

### Impact of resection extent

Finally, we examined whether resection extent contributed to postoperative memory outcome to exclude a potential confounding effect on the observed MC–MQ associations. In patients with LTLE, no significant correlation was found between changes in immediate verbal MQ and the extent of temporal lobe resection (*r* = −0.1210, *P* = 0.5318) or hippocampal resection (*r* = 0.1265, *P* = 0.5131). Similarly, for patients with RTLE, no significant correlation emerged between changes in immediate visual MQ and the extent of temporal lobe resection (*r* = −0.0196*, P* = 0.9404) or hippocampal resection (*r* = 0.0362*, P* = 0.8904). These results suggest that cognitive outcomes measured by MQ were not significantly influenced by the extent of surgical resections in our cohort. Complete results of resection lengths are provided in Supplementary Table 2.

## Discussion

Analysis of MC and memory in MTLE with HS provides insight into the network-level mechanisms underlying cognitive outcomes after epilepsy surgery. By integrating detailed presurgical and postsurgical neuropsychological testing, MRI-based measures of resection extent, and FDG-PET-derived MC, we can identify network-level determinants of memory change. MC alterations can reflect different underlying processes depending on their direction and network context. Typically, decreased MC reflects underlying neuronal dysfunction, inhibitory mechanisms, or long-term network depression due to epileptogenic activity, while increased MC may reflect compensatory or, in some cases, maladaptive network recruitment.^5,6,8,10,12,21,26^

### Presurgical memory correlations

In the presurgical analyses, we found an asymmetry between RTLE and LTLE. In RTLE, higher immediate visual memory scores correlated positively with MC across widespread fronto–cingulate–temporal networks, including the DLPFC, cingulate cortex, temporal lateral cortex, and mesiotemporal regions with frequent involvement of the lesional hippocampus. A considerable proportion of these were cross-hemispheric, particularly between the lesional hippocampus and contralateral DLPFC and temporal neocortex, and additional contralateral connections were identified, including those involving the contralesional hippocampus. This pattern corresponds with fMRI-FC studies indicating that preserved memory in RTLE is supported by recruitment of contralateral and extratemporal regions, suggesting a greater capacity for functional reorganization.^7-10,16,24,32,33^ Overall, these network changes were more extensive in RTLE than in LTLE, consistent with previous MC and FDG-PET studies showing that right-sided MTLE exhibits widespread extratemporal metabolic abnormalities, which may partially explain the wider presurgical correlations observed in our study.^4,13^

In LTLE, immediate verbal memory was exclusively associated with negative correlations, predominantly across cross-hemispheric and extratemporal connections. These involved the temporal lateral cortex, cingulate cortex, lateral parietal cortex, and temporal pole, but not the lesional hippocampus. This finding is consistent with prior fMRI-FC evidence of reduced contralateral involvement and impaired interhemispheric integration in LTLE, reflecting a limited capacity for adaptive network reorganization, potentially influenced by the strong left hemispheric specialization of verbal memory.^10,33,34^ At a conceptual level, this asymmetry is consistent with neuropsychological evidence indicating relatively robust left hemispheric specialization for verbal episodic memory, particularly within the temporal lobe, whereas hemispheric correlates of visual memory are less consistently defined and visual memory processing in right temporal lobe epilepsy has been shown to involve more bilaterally distributed activation patterns, questioning simple material-specific models of memory organization.^10,22,24,35^

### Postsurgical memory correlations

In RTLE, presurgical MC correlated with postsurgical change in immediate visual memory across widespread fronto–cingulate–temporal networks. Most associations were negative, involving the lesional hippocampus together with the cingulate cortex, DLPFC, insula, and temporal neocortex. Negative correlations indicate that patients with higher presurgical MC in these pathways experienced greater postsurgical decline, suggesting that connectivity between the lesional hippocampus and cingulum may have supported memory performance presurgically but was disrupted by AMTR. Similar findings have been reported in fMRI-FC studies showing that reliance on ipsilateral mesial temporal and frontal connectivity is associated with greater postsurgical decline, and with reports of hippocampal–cingulate hyperconnectivity as a maladaptive pattern linked to impaired outcome.^10,24,33^

By contrast, positive correlations were less frequent and localized to contralateral posterior cingulate pathways, involving connections with frontal and temporal regions. In these networks, stronger presurgical MC predicted better postsurgical memory, consistent with prior evidence that contralateral posterior cingulate connectivity with frontal and temporal neocortex supports adaptive reorganization.^7,33^

Whereas presurgical performance in RTLE was associated with networks involving the lesional hippocampus, reliance on these ipsilateral hippocampal–cingulate pathways predicted greater decline after resection, while contralateral posterior cingulate connectivity was associated with preservation. These findings support a distinction between maladaptive and compensatory MC patterns. Increased reliance on ipsilateral hippocampal–cingulate pathways may reflect maladaptive dependence on hippocampus destined for resection, whereas stronger contralateral posterior cingulate connectivity appears to support adaptive reorganization and memory preservation.

In LTLE, no significant associations were identified between presurgical MC and postsurgical memory change, although postsurgical memory decline was more frequent in LTLE. This dissociation may reflect that alterations in MC are generally less pronounced in LTLE and therefore do not yield detectable correlations. Moreover, presurgical correlations in LTLE were exclusively negative, suggesting a maladaptive pattern of network engagement, in which increased extratemporal or contralateral connectivity was associated with poorer memory performance rather than compensation. The left hemisphere also shows a generally lower capacity for contralateral or cross-modal recruitment, further limiting adaptive plasticity. In contrast, in RTLE, visual memory tasks frequently elicit both visual and verbal encoding strategies, engaging bilateral regions.^10,24,36,37^ Such cross-modal recruitment may therefore support compensatory reorganization in right TLE.

### Context for surgical planning

Resection extent did not significantly correlate with cognitive outcome, indicating that MC–memory associations were independent of surgical length in our cohort. However, these results should be interpreted with caution, as we applied a modified methodology and resections in our series were generally shorter, warranting further validation in cohorts with more extensive resections.^6^ Presurgical MC may help to identify patients predisposed to greater postsurgical memory decline and could be incorporated into multimodal approaches for surgical planning, with potential relevance not only for predicting seizure outcome but also for cognitive prognosis.^7,10,24,33,38^

### Limitations and future directions

The main limitations are the smaller postsurgical subgroup size and the 1-year follow-up period. Future studies should utilize larger, multicentre cohorts with advanced analytical methods, and longitudinal assessments extending beyond 1 year. Integrating structural and FC measures (fMRI, EEG, SEEG, diffusion-weighted imaging) is crucial for a comprehensive understanding of networks in MTLE. Although visual MRI ratings in our study showed high inter-rater agreement, automated volumetry may provide a more objective quantitative measure of hippocampal integrity and should be considered in future validation studies.

From a clinical perspective, FDG-PET-derived MC may serve as an adjunctive tool for individualized cognitive risk stratification before AMTR. Because FDG-PET is routinely used in the presurgical evaluation of DRE, MC could be derived from existing clinical imaging data without requiring additional imaging. Semiquantitative and automated PET analyses are increasingly used to complement visual interpretation when supported by appropriate quality control and normative reference data; MC could represent one such quantitative network-based extension.^11,13,14,16-18^ This is relevant because temporal lobe surgery is not only a focal resection but also an intervention within a distributed brain network, and postoperative cognitive outcome likely depends on the integrity and reorganization of remaining memory networks rather than on resection extent alone.^5,7,9,10,38^ In a routine presurgical workflow, ROI-based MC matrices could be generated after standard PET preprocessing, compared with normative reference data, and summarized as patient-level abnormalities in memory-related connectivity patterns. If prospectively validated in multicentre cohorts with prespecified cognitive endpoints, such information could help identify patients at increased risk of postoperative memory decline, guide individualized counselling, select patients for additional memory fMRI or other centre-specific cognitive risk assessments, and support targeted postoperative neuropsychological follow-up.^9,24,39^

## Conclusion

This study demonstrates that MC is related to memory performance in MTLE-HS. In RTLE, presurgical MC involved hippocampal–cingulate, fronto-temporal, and posterior cingulate pathways, which were linked to both presurgical performance and postsurgical outcome. In LTLE, presurgical correlations with memory were confined to lateral temporal, parietal, and cingulate regions, but did not predict postsurgical change. These findings highlight a lateralized dissociation: RTLE was characterized by contralateral recruitment and greater extratemporal involvement, whereas LTLE showed exclusively negative correlations, indicative of maladaptive connectivity and limited potential for reorganization. FDG-PET-derived MC may therefore represent a clinically relevant biomarker for presurgical cognitive risk assessment, particularly in RTLE.

## Supplementary Material

fcag365_Supplementary_Data

## Data Availability

The data that support the findings of this study are not publicly available due to privacy and ethical restrictions related to patient confidentiality. Derived, anonymized data (ROI-based MC matrices) are available from the corresponding author upon reasonable request. The custom Python library developed for calculating individual MC from FDG-PET images was implemented based on the methodology by Huang *et al*.^19^ and is publicly available at https://github.com/Pavel-IO/metabolic-connectivity.

## References

[1] Malmgren K, Thom M. Hippocampal sclerosis—Origins and imaging. Epilepsia. 2012;53(s4):19–33. 10.1111/j.1528-1167.2012.03610.x 22946718

[2] Mathon B, Bielle F, Samson S, et al Predictive factors of long-term outcomes of surgery for mesial temporal lobe epilepsy associated with hippocampal sclerosis. Epilepsia. 2017;58(8):1473–1485. 10.1111/epi.13831 28656696

[3] Cho KH, Lee HJ, Heo K, Kim SE, Lee DA, Park KM. Intrinsic thalamic network in temporal lobe epilepsy with hippocampal sclerosis according to surgical outcomes. Front Neurol. 2021;12:721610. 10.3389/fneur.2021.721610 PMC8429827 34512532

[4] Strýček O, Říha P, Kojan M, Řehák Z, Brázdil M. Metabolic connectivity as a predictor of surgical outcome in mesial temporal lobe epilepsy. Epilepsia Open. 2024;9(1):187–199. 10.1002/epi4.12853 PMC10839369 37881152

[5] Sainburg LE, Englot DJ, Morgan VL. The impact of resective epilepsy surgery on the brain network: Evidence from post-surgical imaging. Brain. 2025;148(6):1866–1875. 10.1093/brain/awaf026 PMC12129734 39854170

[6] Sone D, Ahmad M, Thompson PJ, et al Optimal surgical extent for memory and seizure outcome in temporal lobe epilepsy. Ann Neurol. 2022;91(1):131–144. 10.1002/ana.26266 PMC8916104 34741484

[7] Fleury M, Buck S, Binding LP, et al Episodic memory network connectivity in temporal lobe epilepsy. Epilepsia. 2022;63(10):2597–2622. 10.1111/epi.17370 PMC9804196 35848050

[8] Sidhu MK, Stretton J, Winston GP, et al A functional magnetic resonance imaging study mapping the episodic memory encoding network in temporal lobe epilepsy. Brain. 2013;136(6):1868–1888. 10.1093/brain/awt099 PMC3673458 23674488

[9] Sidhu MK, Stretton J, Winston GP, et al Memory fMRI predicts verbal memory decline after anterior temporal lobe resection. Neurology. 2015;84(15):1512–1519. 10.1212/WNL.0000000000001461 PMC4408284 25770199

[10] Sidhu MK, Stretton J, Winston GP, et al Memory network plasticity after temporal lobe resection: A longitudinal functional imaging study. Brain. 2016;139(2):415–430. 10.1093/brain/awv365 PMC4805088 26754787

[11] Guedj E, Varrone A, Boellaard R, et al EANM procedure guidelines for brain PET imaging using [18F]FDG, version 3. Eur J Nucl Med Mol Imaging. 2022;49(2):632–651. 10.1007/s00259-021-05603-w PMC8803744 34882261

[12] Logothetis NK . What we can do and what we cannot do with fMRI. Nature. 2008;453(7197):869–878. 10.1038/nature06976 18548064

[13] Chassoux F, Artiges E, Semah F, et al ^ 18 ^ F-FDG-PET patterns of surgical success and failure in mesial temporal lobe epilepsy. Neurology. 2017;88(11):1045–1053. 10.1212/WNL.0000000000003714 28188304

[14] Choi JY, Kim SJ, Hong SB, et al Extratemporal hypometabolism on FDG PET in temporal lobe epilepsy as a predictor of seizure outcome after temporal lobectomy. Eur J Nucl Med Mol Imaging. 2003;30(4):581–587. 10.1007/s00259-002-1079-8 12557048

[15] Griffith HR, Perlman SB, Woodard AR, et al Preoperative FDG-PET temporal lobe hypometabolism and verbal memory after temporal lobectomy. Neurology. 2000;54(5):1161–1165. 10.1212/wnl.54.5.1161 10720291

[16] Kamm J, Boles Ponto LL, Manzel K, et al Temporal lobe asymmetry in FDG-PET uptake predicts neuropsychological and seizure outcomes after temporal lobectomy. Epilepsy Behav. 2018;78:62–67. 10.1016/j.yebeh.2017.10.006 PMC6585418 29175222

[17] Güvenç C, Dupont P, Van Den Stock J, et al Correlation of neuropsychological and metabolic changes after epilepsy surgery in patients with left mesial temporal lobe epilepsy with hippocampal sclerosis. EJNMMI Res. 2018;8(1):31. 10.1186/s13550-018-0385-5 PMC5897268 29651571

[18] Leeman BA, Leveroni CL, Johnson KA. Does hippocampal FDG-PET asymmetry predict verbal memory dysfunction after left temporal lobectomy? Epilepsy Behav. 2009;16(2):274–280. 10.1016/j.yebeh.2009.07.035 19726233

[19] Huang SY, Hsu JL, Lin KJ, Hsiao IT. A novel individual metabolic brain network for 18F-FDG PET imaging. Front Neurosci. 2020;14:344. 10.3389/fnins.2020.00344 PMC7235322 32477042

[20] Wechsler D . Wechsler Memory Scale. 3rd ed. Czech experimental version. Psychodiagnostika s.r.o.; 1999.

[21] Chassoux F . Metabolic changes and electro-clinical patterns in mesio-temporal lobe epilepsy: A correlative study. Brain. 2004;127(1):164–174. 10.1093/brain/awh014 14534161

[22] Binder JR, Desai RH, Graves WW, Conant LL. Where is the semantic system? A critical review and meta-analysis of 120 functional neuroimaging studies. Cereb Cortex. 2009;19(12):2767–2796. 10.1093/cercor/bhp055 PMC2774390 19329570

[23] Blumenfeld RS, Ranganath C. Prefrontal Cortex and long-term memory encoding: An integrative review of findings from neuropsychology and neuroimaging. Neuroscientist. 2007;13(3):280–291. 10.1177/1073858407299290 17519370

[24] Bonelli SB, Powell RHW, Yogarajah M, et al Imaging memory in temporal lobe epilepsy: Predicting the effects of temporal lobe resection. Brain. 2010;133(4):1186–1199. 10.1093/brain/awq006 PMC2850579 20157009

[25] Stoeckel C, Gough PM, Watkins KE, Devlin JT. Supramarginal gyrus involvement in visual word recognition. Cortex. 2009;45(9):1091–1096. 10.1016/j.cortex.2008.12.004 PMC2726132 19232583

[26] Vilberg KL, Rugg MD. Memory retrieval and the parietal cortex: A review of evidence from a dual-process perspective. Neuropsychologia. 2008;46(7):1787–1799. 10.1016/j.neuropsychologia.2008.01.004 PMC2488316 18343462

[27] Hsiao IT, Huang CC, Hsieh CJ, et al Perfusion-like template and standardized normalization-based brain image analysis using 18F-florbetapir (AV-45/Amyvid) PET. Eur J Nucl Med Mol Imaging. 2013;40(6):908–920. 10.1007/s00259-013-2350-x 23412134

[28] Aggleton JP, O’Mara SM. The anterior thalamic nuclei: Core components of a tripartite episodic memory system. Nat Rev Neurosci. 2022;23(8):505–516. 10.1038/s41583-022-00591-8 35478245

[29] He X, Doucet GE, Pustina D, Sperling MR, Sharan AD, Tracy JI. Presurgical thalamic ‘hubness’ predicts surgical outcome in temporal lobe epilepsy. Neurology. 2017;88(24):2285–2293. 10.1212/WNL.0000000000004035 PMC12477986 28515267

[30] Bonnici HM, Sidhu M, Chadwick MJ, Duncan JS, Maguire EA. Assessing hippocampal functional reserve in temporal lobe epilepsy: A multi-voxel pattern analysis of fMRI data. Epilepsy Res. 2013;105(1-2):140–149. 10.1016/j.eplepsyres.2013.01.004 PMC3682189 23352740

[31] Bell B, Hermann B, Seidenberg M. Significant discrepancies between immediateand delayed WMS-III indices are rarein temporal lobe epilepsy patients. Clin Neuropsychol. 2004;18(2):303–311. 10.1080/13854040490501646 PMC1255965 15587676

[32] Alessio A, Pereira FRS, Sercheli MS, et al Brain plasticity for verbal and visual memories in patients with mesial temporal lobe epilepsy and hippocampal sclerosis: An fMRI study. Hum Brain Mapp. 2013;34(1):186–199. 10.1002/hbm.21432 PMC6870348 22038783

[33] Doucet GE, Skidmore C, Evans J, et al Temporal lobe epilepsy and surgery selectively Alter the dorsal, not the ventral, default-mode network. Front Neurol. 2014;5:23. 10.3389/fneur.2014.00023 PMC3948047 24653713

[34] Zhao X, Kang H, Zhou Z, et al Interhemispheric functional connectivity asymmetry is distinctly affected in left and right mesial temporal lobe epilepsy. Brain Behav. 2022;12(3):e2484. 10.1002/brb3.2484 PMC8933759 35166072

[35] Baxendale S, Thompson P. Beyond localization: The role of traditional neuropsychological tests in an age of imaging. Epilepsia. 2010;51(11):2225–2230. 10.1111/j.1528-1167.2010.02710.x 21175602

[36] Kelley WM, Miezin FM, McDermott KB, et al Hemispheric specialization in human dorsal frontal Cortex and medial temporal lobe for verbal and nonverbal memory encoding. Neuron. 1998;20(5):927–936. 10.1016/s0896-6273(00)80474-2 9620697

[37] Diaz-Orueta U, Rogers BM, Blanco-Campal A, Burke T. The challenge of neuropsychological assessment of visual/visuo-spatial memory: A critical, historical review, and lessons for the present and future. Front Psychol. 2022;13:962025. 10.3389/fpsyg.2022.962025 PMC9447442 36081731

[38] Fleury MN, Binding LP, Taylor P, et al Long-term memory plasticity in a decade-long connectivity study post anterior temporal lobe resection. Nat Commun. 2025;16(1):692. 10.1038/s41467-024-55704-x PMC11735635 39814723

[39] Szaflarski JP, Gloss D, Binder JR, et al Practice guideline summary: Use of fMRI in the presurgical evaluation of patients with epilepsy: Report of the guideline development, dissemination, and implementation subcommittee of the American academy of neurology. Neurology. 2017;88(4):395–402. 10.1212/WNL.0000000000003532 PMC5272968 28077494

