# Supplementary material for: Metabolic connectivity and memory in mesial temporal lobe epilepsy
Source: Brain Commun. 2026 Oct 14;8(5):fcag365. doi: 10.1093/braincomms/fcag365 (PMC13663807; doi:10.1093/braincomms/fcag365)
Supplement: Supplementary file 1 [file fcag365_supplementary_data.docx]

# **Supplementary** **material**

**Regions of interest**

The analysis focused on a set of cortical and subcortical hubs implicated in memory processing, with particular relevance in TLE. These included:

- Temporal lobe structures. These included the lateral temporal cortex (superior, middle, and inferior temporal gyri) and temporal pole to capture hippocampal interactions and long-term memory processes. The lateral temporal cortex participates in semantic and auditory–verbal processing and connects with frontoparietal and cingulate hubs important for episodic memory^1–3^. The temporal pole acts as an integrative hub linking the hippocampus with semantic and autobiographical memory networks, and shows altered activation and connectivity in TLE associated with memory impairment^1,3,4^. Preserved extra-hippocampal temporal connectivity in these regions has been associated with better episodic memory performance in MTLE^5^.
- Dorsolateral prefrontal cortex (DLPFC). DLPFC supports executive functions essential for memory encoding and retrieval, and middle frontal gyrus connectivity has been associated with preserved memory performance and reduced postoperative decline in MTLE^3,6^.
- Lateral parietal cortex/lobulus parietalis inferior. This area contributes to multimodal integration, phonological working memory, and verbal processing^7,8^.
- Insula. The insula is involved in mesial temporal seizure propagation and acts as an extra-hippocampal hub for memory integration, with preserved hippocampo–insular connectivity and adaptive recruitment linked to better cognitive outcomes in MTLE^5,9^.
- Cingulate gyrus. The cingulate gyrus is a key part of the memory network, where the posterior cingulate gyrus plays a role in episodic memory and emotional processing. It has strong reciprocal connections with the hippocampal complex. The anterior cingulate gyrus, as part of the ventromedial prefrontal cortex, is associated with motivation and the affective processing of concepts^1^. Contralateral connectivity is linked to better outcomes, whereas persistent ipsilateral hippocampal–cingulate coupling can be maladaptive^4,5^.
- Fusiform gyrus. The fusiform gyrus supports perceptual–mnemonic integration of complex visual stimuli and contributes to visual aspects of episodic memory. In TLE, fusiform–hippocampal connectivity is reorganized, and preserved fusiform involvement has been linked to better memory performance^5,10^.
- Orbitofrontal cortex (OFC). The OFC contributes to episodic memory by supporting encoding and retrieval of temporal context. Functional MRI and longitudinal connectivity studies demonstrate involvement of medial orbitofrontal and gyrus rectus regions in memory networks affected by temporal lobe resection^5^.
- Thalamus. The thalamus, particularly the anterior nuclei, is a central component of the hippocampal–diencephalic episodic memory system. Given its role in episodic memory and its altered network connectivity in TLE, including presurgical thalamic network hubness associated with surgical outcome, the thalamus was included as a relevant subcortical hub^11,12^.

The complete ROI set, defined according to the AAL atlas, included the hippocampus, fusiform gyrus, temporal pole, lateral temporal cortices (superior, middle, and inferior gyri), dorsolateral prefrontal cortex (superior and middle frontal gyri), orbitofrontal cortex (rectus gyri), insula, angular and supramarginal gyri, anterior and posterior cingulate cortex, and thalamus. The AAL labels used in the figures are listed below (L – left; R – right).

- Frontal_Sup_L/R – Superior frontal gyrus
- Frontal_Mid_L/R – Middle frontal gyrus
- Insula_L/R – Insula
- Cingulum_Ant_L/R – Anterior cingulate gyrus
- Cingulum_Post_L/R – Posterior cingulate gyrus
- Rectus_L/R – Gyrus rectus
- Hippocampus_L/R – Hippocampus
- Fusiform_L/R – Fusiform gyrus
- Angular_L/R – Angular gyrus
- SupraMarginal_L/R – Supramarginal gyrus
- Temporal_Sup_L/R – Superior temporal gyrus
- Temporal_Mid_L/R – Middle temporal gyrus
- Temporal_Inf_L/R – Inferior temporal gyrus
- Temporal_Pole_Sup_L/R – Superior temporal pole
- Thalamus_L/R – Thalamus

### **Supplementary Figure 1. Correlation of metabolic connectivity with postsurgical memory change in left temporal lobe epilepsy**

Correlation matrix showing the absence of significant associations between metabolic connectivity and postsurgical change in immediate verbal memory in left temporal lobe epilepsy**.** Positive values indicate improvement in memory after surgery. Statistical analysis was performed using Pearson correlation across individual LTLE patients with available postsurgical testing (n = 29), followed by false discovery rate correction across connections. The lower left triangle depicts Pearson r values, and the upper right triangle indicates statistically significant results (FDR-corrected p < 0.05). No connections survived FDR correction. Abbreviations: L – left; R – right; MC – metabolic connectivity; MQ – memory quotient; FDR – false discovery rate; LTLE – left temporal lobe epilepsy. Abbreviations of anatomical regions are provided above.

### **Supplementary Table 1. Correlations of metabolic connectivity and memory**

| **Analysis** | **p** | **r** | **d** | **ROI1** | **ROI2** |
| --- | --- | --- | --- | --- | --- |
| **RTLE presurgical (MC and immediate visual MQ correlation)** *n=39* | 0.00001125 | 0.6473 | -0.0905 | Cingulum_Ant_L | Frontal_Sup_L |
|  | 0.00004184 | 0.6137 | -0.157 | Cingulum_Ant_L | Frontal_Sup_R |
|  | 0.00000281 | 0.6788 | -0.0399 | Cingulum_Ant_L | Frontal_Mid_L |
|  | 0.00269094 | 0.4733 | -0.1537 | Cingulum_Ant_L | Frontal_Mid_R |
|  | 0.00021082 | 0.5663 | 0.0111 | Cingulum_Post_R | Frontal_Sup_R |
|  | 0.00000294 | 0.6778 | 0.0189 | Cingulum_Post_R | Frontal_Mid_L |
|  | 0.00019286 | 0.5691 | -0.0016 | Cingulum_Post_R | Frontal_Mid_R |
|  | 0.00050538 | 0.5372 | 0.024 | Hippocampus_L | Frontal_Mid_L |
|  | 0.00189691 | 0.4877 | 0.0164 | Hippocampus_R | Frontal_Mid_L |
|  | 0.00052718 | 0.5358 | 0.0135 | Hippocampus_R | Frontal_Mid_R |
|  | 0.00127969 | 0.5032 | -0.0203 | Hippocampus_R | Insula_L |
|  | 0.0013843 | 0.5002 | -0.0906 | Angular_L | Frontal_Mid_L |
|  | 0.00031812 | 0.5529 | 0.0114 | Angular_R | Hippocampus_R |
|  | 0.00273535 | 0.4726 | -0.0055 | Temporal_Sup_L | Hippocampus_L |
|  | 0.0012831 | 0.5031 | 0.0121 | Temporal_Sup_L | Hippocampus_R |
|  | 0.00214175 | 0.4828 | -0.008 | Temporal_Sup_R | Hippocampus_R |
|  | 0.00036926 | 0.548 | -0.0161 | Temporal_Pole_Sup_L | Frontal_Sup_R |
|  | 0.00136313 | 0.5008 | 0.0013 | Temporal_Pole_Sup_L | Frontal_Mid_L |
|  | 0.00045728 | 0.5407 | -0.0602 | Temporal_Pole_Sup_L | Frontal_Mid_R |
|  | 0.00036317 | 0.5485 | -0.0055 | Temporal_Pole_Sup_R | Hippocampus_R |
|  | 0.00222824 | 0.4811 | 0.031 | Temporal_Inf_R | Frontal_Sup_L |
|  | 0.00019638 | 0.5685 | 0.0002 | Temporal_Inf_R | Frontal_Sup_R |
|  | 0.00056977 | 0.533 | 0.0686 | Temporal_Inf_R | Frontal_Mid_L |
|  | 0.00253203 | 0.4758 | 0.0068 | Temporal_Inf_R | Frontal_Mid_R |
| **LTLE presurgical (MC and immediate verbal MQ correlation)** *n=44* | 0.00024807 | -0.5311 | -0.1485 | Temporal_Sup_R | Cingulum_Ant_L |
|  | 0.00044756 | -0.5121 | -0.0312 | Temporal_Sup_R | Fusiform_R |
|  | 0.00027669 | -0.5277 | -0.0809 | Temporal_Sup_R | Angular_L |
|  | 0.00066739 | -0.4986 | -0.1749 | Temporal_Pole_Sup_L | Temporal_Sup_R |
|  | 0.00037666 | -0.5178 | -0.1328 | SupraMarginal_R | Cingulum_Ant_L |
|  | 0.00055383 | -0.5049 | -0.1303 | SupraMarginal_R | Temporal_Inf_L |
| **RTLE postsurgical (MC and immediate visual MQ correlation)** *n=17* | 0.00155871 | -0.7054 | -0.0536 | Frontal_Sup_R | Frontal_Sup_L |
|  | 0.00011418 | -0.8003 | -0.0004 | Frontal_Mid_R | Frontal_Sup_L |
|  | 0.00024206 | -0.7771 | -0.0124 | Insula_L | Frontal_Sup_L |
|  | 0.0021071 | -0.6915 | -0.0248 | Insula_L | Frontal_Sup_R |
|  | 0.00002983 | -0.8354 | 0.0416 | Rectus_R | Rectus_L |
|  | 0.00212957 | 0.691 | -0.0857 | Cingulum_Post_L | Frontal_Mid_R |
|  | 0.00033579 | -0.7661 | -0.022 | Cingulum_Post_R | Cingulum_Ant_R |
|  | 0.00089959 | -0.729 | 0.0141 | Cingulum_Post_R | Rectus_L |
|  | 0.00035649 | -0.764 | 0.0024 | Hippocampus_L | Rectus_L |
|  | 0.00090268 | -0.7289 | -0.0636 | Hippocampus_R | Frontal_Sup_L |
|  | 0.00088229 | -0.7298 | -0.0457 | Hippocampus_R | Insula_L |
|  | 0.00097695 | -0.7256 | -0.0309 | Hippocampus_R | Insula_R |
|  | 0.00026374 | -0.7743 | -0.0164 | Hippocampus_R | Cingulum_Ant_R |
|  | 0.00002954 | -0.8357 | -0.073 | Angular_L | Insula_L |
|  | 0.00213335 | -0.6909 | -0.0196 | Angular_R | Hippocampus_R |
|  | 0.00132774 | -0.7126 | -0.1671 | Temporal_Sup_R | Cingulum_Ant_L |
|  | 0.00124651 | 0.7153 | -0.0444 | Temporal_Mid_L | Cingulum_Post_L |
|  | 0.00015434 | -0.7914 | -0.2447 | Temporal_Mid_R | Cingulum_Ant_L |
|  | 0.00073552 | -0.7371 | -0.1341 | Temporal_Inf_R | Temporal_Sup_R |
|  | 0.00057226 | -0.7468 | -0.1125 | Temporal_Inf_R | Temporal_Mid_R |

ROI1 and ROI2 indicate the pair of regions of interest between which the correlation was calculated. Abbreviations: RTLE – right temporal lobe epilepsy; LTLE – left temporal lobe epilepsy; MC – metabolic connectivity; MQ – memory quotient; p – p-value; r – correlation coefficient; d – mean difference from control group; ROI1/2 – regions of interest.

### **Supplementary Table 2. Extent of hippocampal and temporal lobe resection**

| **Group** | **Total hippocampal length (mm), mean ± SD (range)** | **Resected hippocampal length (mm), mean ± SD (range)** | **% hippocampal resection, mean (range)** | **Total temporal lobe length (mm), mean ± SD (range)** | **Resected temporal lobe length (mm), mean ± SD (range)** | **% temporal lobe resection, mean (range)** |
| --- | --- | --- | --- | --- | --- | --- |
| **LTLE** | 42.9 ± 6.5 | 13.9 ± 4.2 | 32% | 75.3 ± 2.2 | 33.5 ± 8.7 | 44% |
| **RTLE** | 44.8 ± 2.8 | 16.1 ± 4.1 | 36% | 76.2 ± 2.4 | 34.5 ± 6.5 | 45% |
| **All TLE** | 43.8 ± 5.1 | 14.9 ± 4.3 | 34% | 75.7 ± 2.3 | 34.0 ± 7.7 | 45% |

All measurements were performed in MNI space following the methodology of Sone et al. ^6^. The anterior end of the temporal lobe and posterior hippocampal tail were identified, the hippocampal axis was defined, and total and resected lengths of both structures were measured along this axis. Abbreviations: RTLE – right temporal lobe epilepsy; LTLE – left temporal lobe epilepsy.

**Supplementary references**

1. Binder, J. R., Desai, R. H., Graves, W. W., & Conant, L. L. (2009). Where is the semantic system? A critical review and meta-analysis of 120 functional neuroimaging studies. Cerebral Cortex, 19(12), 2767–2796. <https://doi.org/10.1093/cercor/bhp055>
2. Doucet, G. E., Skidmore, C., Evans, J., et al. (2014). Temporal lobe epilepsy and surgery selectively alter the dorsal, not the ventral, default-mode network. Frontiers in Neurology, 5. <https://doi.org/10.3389/fneur.2014.00023>
3. Bonnici, H. M., Sidhu, M., Chadwick, M. J., Duncan, J. S., & Maguire, E. A. (2013). Assessing hippocampal functional reserve in temporal lobe epilepsy: A multi-voxel pattern analysis of fMRI data. Epilepsy Research, 105(1–2), 140–149. <https://doi.org/10.1016/j.eplepsyres.2013.01.004>
4. Sidhu, M. K., Stretton, J., Winston, G. P., et al. (2015). Memory fMRI predicts verbal memory decline after anterior temporal lobe resection. Neurology, 84(15), 1512–1519. <https://doi.org/10.1212/WNL.0000000000001461>
5. Fleury, M., Buck, S., Binding, L. P., et al. (2022). Episodic memory network connectivity in temporal lobe epilepsy. Epilepsia, 63(10), 2597–2622. <https://doi.org/10.1111/epi.17370>
6. Blumenfeld, R. S., & Ranganath, C. (2007). Prefrontal cortex and long-term memory encoding: An integrative review of findings from neuropsychology and neuroimaging. The Neuroscientist, 13(3), 280–291. <https://doi.org/10.1177/1073858407299290>
7. Stoeckel, C., Gough, P. M., Watkins, K. E., & Devlin, J. T. (2009). Supramarginal gyrus involvement in visual word recognition. Cortex, 45(9), 1091–1096. <https://doi.org/10.1016/j.cortex.2008.12.004>
8. Vilberg, K. L., & Rugg, M. D. (2008). Memory retrieval and the parietal cortex: A review of evidence from a dual-process perspective. Neuropsychologia, 46(7), 1787–1799. <https://doi.org/10.1016/j.neuropsychologia.2008.01.004>
9. Sidhu, M. K., Stretton, J., Winston, G. P., et al. (2013). A functional magnetic resonance imaging study mapping the episodic memory encoding network in temporal lobe epilepsy. Brain, 136(6), 1868–1888. <https://doi.org/10.1093/brain/awt099>
10. Fleury, M. N., Binding, L. P., Taylor, P., et al. (2025). Long-term memory plasticity in a decade-long connectivity study post anterior temporal lobe resection. Nature Communications, 16(1), 692. <https://doi.org/10.1038/s41467-024-55704-x>
11. Aggleton, J. P., & O’Mara, S. M. (2022). The anterior thalamic nuclei: Core components of a tripartite episodic memory system. Nature Reviews Neuroscience, 23(8), 505–516. <https://doi.org/10.1038/s41583-022-00591-8>
12. He, X., Doucet, G. E., Pustina, D., Sperling, M. R., Sharan, A. D., & Tracy, J. I. (2017). Presurgical thalamic “hubness” predicts surgical outcome in temporal lobe epilepsy. Neurology, 88(24), 2285–2293. <https://doi.org/10.1212/WNL.0000000000004035>
